# Thyroid intelligent diagnosis based on THMSNet

**DOI:** 10.3389/fendo.2025.1686248

**Published:** 2025-12-05

**Authors:** Zhen Rao, Tao Yu, Xitan Yu

**Affiliations:** 1 Changde Hospital, Xiangya School of Medicine, Central South University, The First People’s Hospital of Changde City, Changde, China; 2 Xinjiang Medical University, Ürümqi, China

**Keywords:** thyroid nodule diagnosis, deep learning, multiscale feature extraction, Mamba architecture, attention mechanism, probability calibration, clinical decision support

## Abstract

**Background:**

Thyroid disease is a common endocrine disorder, with the differentiation between benign and malignant nodules being critical for clinical decision-making. Traditional diagnostic methods, such as ultrasound and TI-RADS classification, are limited by interobserver variability and time-consuming processes. While deep learning approaches such as CNNs and transformers have shown promise, they face challenges in multiscale feature extraction, global dependency modeling, and alignment with clinical standards.

**Methods:**

We proposes THMSNet, a hybrid architecture that integrates a pyramid structure for multiscale feature extraction and Mamba for global long-range dependency modeling. The serial channel–spatial attention module (SCSAM) enhances feature representation, whereas the truth–value calibration (TVC) algorithm aligns model predictions with pathological standards. The system is evaluated on a public dataset of 7,288 thyroid ultrasound images (3,282 benign, 4,006 malignant) via five metrics: accuracy, precision, recall, F1 score, and AUROC.

**Results:**

THMSNet achieves 91.15% accuracy, 93.28% recall, and 96.92% AUROC, outperforming ResNet (86.03% accuracy) and DenseNet (95.50% AUROC). Confidence intervals are calculated for key metrics, further strengthening the rigor of results. Ablation studies confirm the utility of each module, with the pyramid architecture (+7.83% accuracy), Mamba (+2.99%), SCSAM (+6.94%), and TVC (+6.94%) progressively contributing to performance improvements.

**Conclusion:**

THMSNet provides a robust and clinically applicable solution for thyroid nodule diagnosis, combining advanced feature extraction, attention mechanisms, and probability calibration. Its high accuracy and interpretability make it a valuable tool for assisting radiologists in clinical practice.

## 1 Introduction

Thyroid disease is a common endocrine system disorder in which the differentiation between benign and malignant thyroid nodules is particularly critical. Studies have shown that approximately 5–15% of thyroid nodules carry a risk of malignancy (1). These nodules exhibit complex characteristics on ultrasound imaging: diverse morphology (solid, cystic, or mixed), varying sizes (ranging from a few millimeters to several centimeters), and variable boundary features (malignant nodules often present with irregular margins) (2). Currently, clinical diagnosis relies primarily on ultrasound examination and the TI-RADS classification system for manual evaluation (3), but this approach has significant limitations: diagnostic results are highly influenced by physician experience (with interobserver variability exceeding 20%) (4), the evaluation process is time-consuming (10–15 minutes per case) (5), and the recognition rate for subtle features is low (6). Although computer-aided diagnostic methods based on manual feature extraction (such as gray-level co-occurrence matrix) and machine learning (e.g., SVM) have been developed, their AUC typically remains below 0.75, particularly showing insufficient sensitivity for small nodules (<1 cm), which significantly limits their clinical utility (7).

Parallel advancements have also occurred in the development of attention mechanisms for medical image analysis within the field of deep learning, primarily focusing on two technical approaches: CNNs and transformers. In CNN research, multiple groundbreaking achievements have demonstrated unique advantages: Wu et al. (8) developed a deep learning system based on ACR TI-RADS that achieved excellent AUC values of 0.904 and 0.845 in differentiating TR4 and TR5 category nodules, respectively, with diagnostic performance significantly surpassing that of experienced radiologists; Chi et al. (9) implemented a fine-tuning strategy using GoogLeNet to achieve 86% classification accuracy; Nugroho et al. (10) systematically evaluated and confirmed that the NasNetLarge model improved accuracy by 8% compared with DenseNet121; Wang et al. (11) developed the ThyroNet-X4 Genesis model, which achieved outstanding performance of 85.55% accuracy on internal training sets. However, CNN methods have a critical limitation: their local receptive field characteristics result in insufficient modeling of global correlations among overall lesion features. To overcome these limitations, researchers have explored transformer architectures: Sun et al. (12) innovatively developed the TC-ViT model, achieving 86.9% accuracy in TI-RADS category 3 nodule classification; Huang et al. (13) designed the SRT model, which demonstrated exceptional comprehensive performance (accuracy 0.8832, AUC 0.8660); Baima et al. (14) validated a dense node Swin-Transformer model using multicenter data from 17 hospitals, achieving 87.27% accuracy; and Zhao et al. (15) recently developed the UTV-ST Swin transformer, which attained 82.1% accuracy in LNM prediction. Nevertheless, transformer-based models often struggle to capture fine-grained local features—such as microcalcifications and irregular margins—due to their coarse-grained tokenization and lack of inductive bias for local structures, which is particularly critical in thyroid ultrasound tasks.

Attention mechanisms have also evolved to enhance feature representation. While squeeze-and-excitation (SE) (16), convolutional block attention module (CBAM) (17), SCSE (18), coordinate attention (CA) (19), and global attention mechanism (GAM) (20) have each contributed to improving channel or spatial focus, they often adopt parallel structures that fail to model the hierarchical dependencies between channel and spatial dimensions, and lack cross-dimensional interaction—a limitation especially pertinent in medical images with hierarchical anatomical structures.

Current algorithms still face challenges regarding model and parameter uncertainty in prediction. Abdullah et al. (21) proposed a ranking-based Bayesian ensemble learning method that significantly improves uncertainty quantification in medical image classification by selecting top-k models. Wei et al. (22) developed an ensemble deep learning model (EDLC-TN) that achieved 98.51% classification accuracy on a multicenter thyroid nodule dataset containing 26,541 images. Bórquez et al. (23) employed a Monte Carlo dropout approach, attaining 0.89 accuracy in classifying HER2-overexpressing breast cancer tissues while effectively identifying high-uncertainty regions. Lakshminarayanan et al. (24) introduced a deep ensemble method that provides a simple and scalable uncertainty estimation solution, outperforming traditional Bayesian neural networks. Benamrane et al. (25) combined fuzzy neural networks with genetic algorithms to offer an interpretable solution for medical image anomaly detection. However, these methods still face challenges such as high computational complexity and poor adaptability to small-sample scenarios, and the discrepancy between model predictions and clinical diagnostic standards remains inadequately addressed, resulting in insufficient interpretability of the results.

To address the aforementioned three challenges—insufficient multiscale feature extraction, weak long-range dependency modeling, and significant deviation between prediction results and clinical standards—this paper proposes THMSNet, with the following innovations:

### 1.1 Hybrid and Mamba architecture

A pyramid structure is employed to extract local multiscale features and is combined with Mamba for modeling global long-range dependencies, achieving more comprehensive feature representation.

### 1.2 SCSAM attention mechanism

A serial channel-spatial attention mechanism is introduced to enhance feature representation in key regions, improving classification accuracy.

### 1.3 Ground truth calibration algorithm

This algorithm aligns model predictions with pathological diagnostic standards, reducing result bias and enhancing clinical applicability.

## 2 Related works

### 2.1 Pyramid architecture

The pyramid architecture has been widely adopted in medical image analysis because of its ability to capture multiscale features, which is crucial for detecting lesions of varying sizes. Notable implementations include the FPN (26), which leverages hierarchical feature maps to improve diagnostic accuracy. These architectures have demonstrated success in tasks such as tumor segmentation and classification, providing a foundation for advanced thyroid nodule analysis.

### 2.2 Mamba architecture

The Mamba architecture, which is based on state space models, has emerged as a powerful tool for modeling long-range dependencies in sequential data, including medical images. Recent studies (27) highlight its efficiency in capturing the global context while maintaining computational scalability. Mamba’s selective state space mechanism has shown promise in enhancing feature representation, making it suitable for complex tasks such as thyroid malignancy detection.

## 3 Methods

### 3.1 Intelligent thyroid diagnosis based on HMSNet

**Figure 1:**
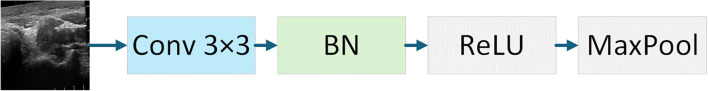
Basic feature extraction.

This study designs a basic feature extraction module (BFEM) that adopts a classic four-stage ‘convolution-normalization-activation-pooling’ cascade structure, as illustrated in **Figure 1**. The module first extracts local texture features of thyroid nodules (such as margin sharpness and internal echogenicity) through a 3×3 convolutional layer, followed by a batch normalization (BN) layer to accelerate model convergence. A ReLU activation function is then applied to introduce nonlinear transformations, and finally, a max pooling layer (MaxPool) is used for feature dimensionality reduction. This design not only ensures computational efficiency but also effectively captures the fundamental morphological characteristics of the nodules, establishing a crucial foundation for subsequent multiscale feature fusion.

**Figure 2:**
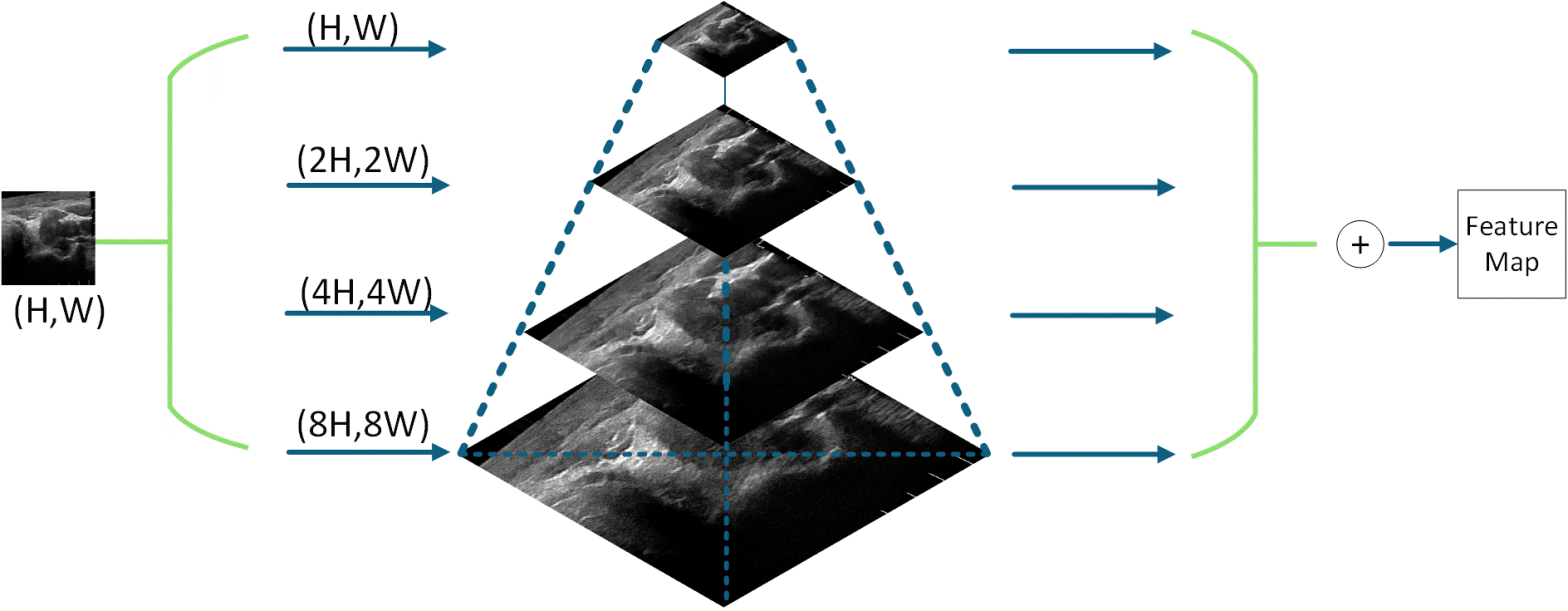
Hierarchical multiscale feature pyramid module.

As illustrated in **Figure 2**, the proposed hierarchical multiscale feature pyramid (HMFP) module constructs a four-level feature pyramid architecture. The base layer at (H,W) resolution captures global semantic information, whereas the intermediate (2H,2 W) layer focuses on the main structural characteristics of lesions. The fine-grained (4H,4 W) layer enhances boundary features, and the high-resolution (8H,8 W) layer preserves crucial details such as microcalcifications. Adaptive feature alignment between different levels is achieved through deformable convolution (Deformable Conv), with skip connections effectively integrating multiscale features.

**Figure 3:**
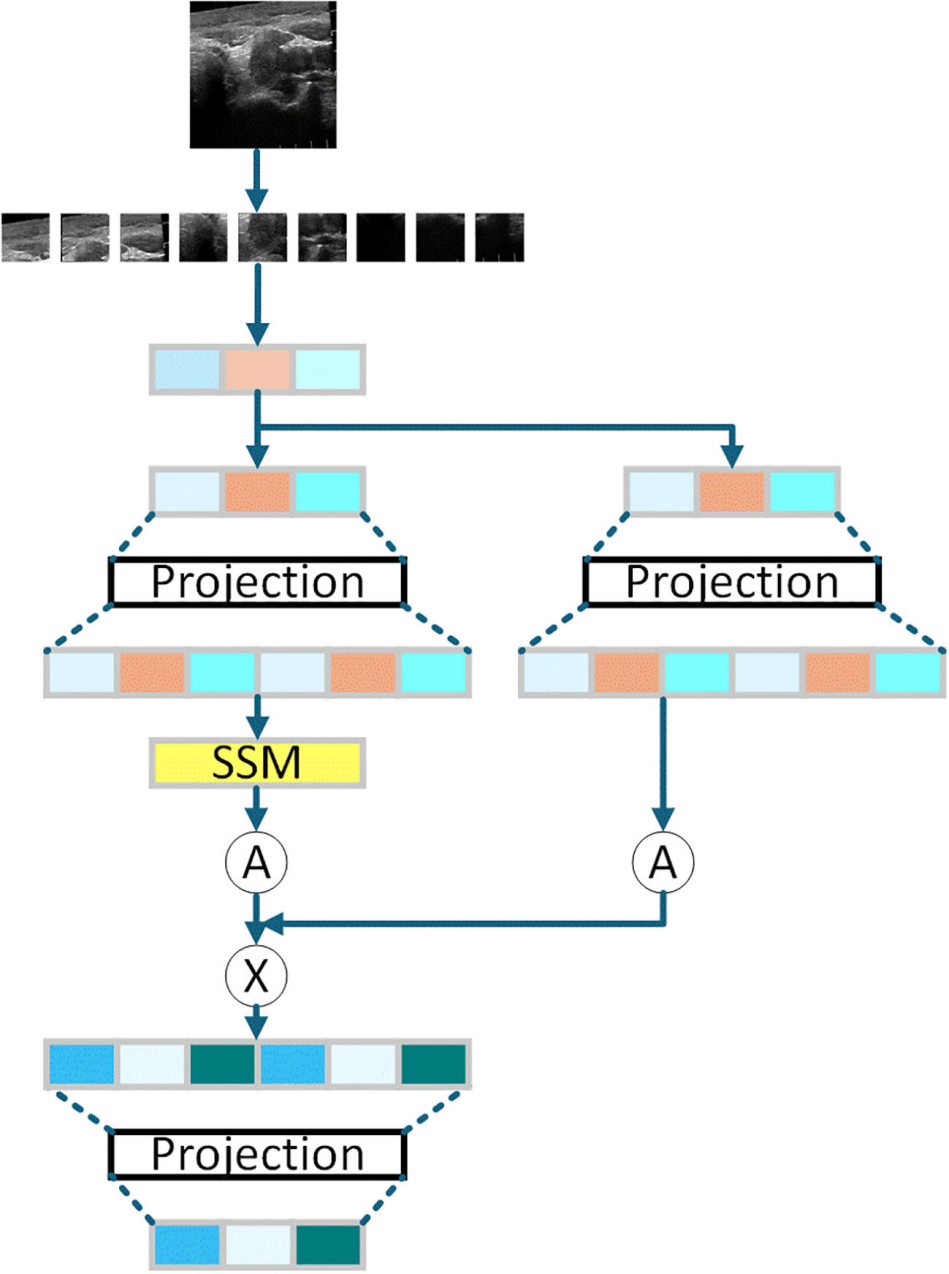
Mamba-based multistage projection module.

The proposed Mamba-based multistage projection module (MMPM) adopts a three-stage “projection-SSM-state update” architecture, as shown in **Figure 3**. In the first stage, features are projected into the latent space through a 1×1 convolution. The Mamba block then performs sequence modeling with a 256-token sequence length in the second stage. Finally, the hidden states are updated via a gating mechanism. Notably, the state space model (SSM) component employs a parameterization mechanism to enable dynamic adjustment of attention weights for key features.

**Figure 4:**
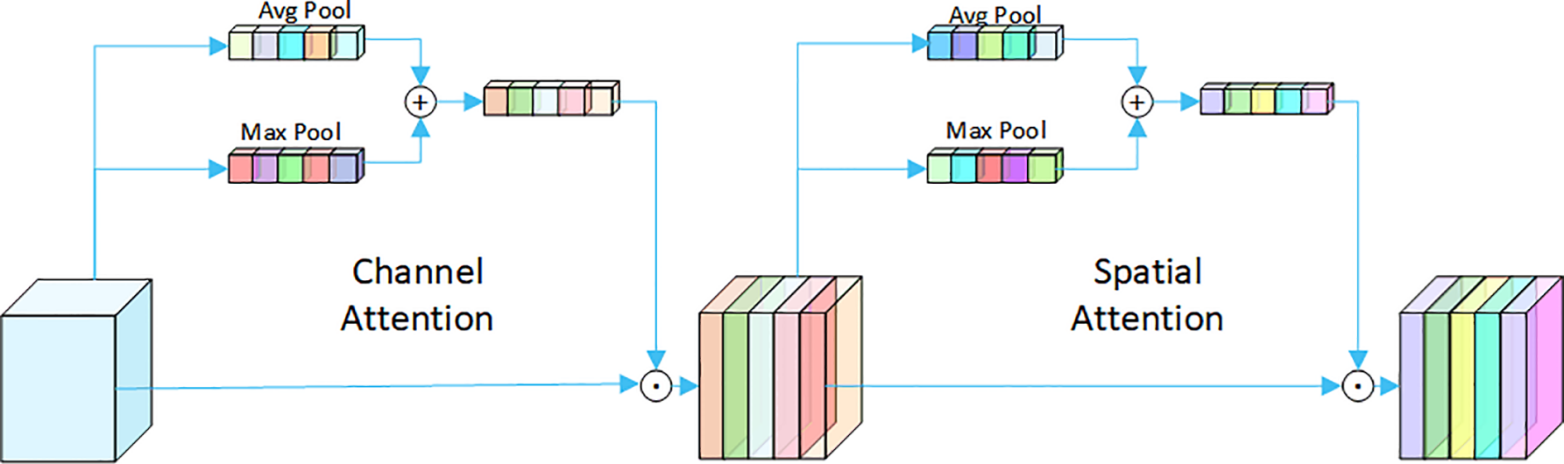
Serial channel-spatial attention module.

The proposed serial channel–spatial attention module (SCSAM), illustrated in **Figure 4**, implements a strict “channel–first” processing pipeline. The module initially computes channelwise weights through a channel attention mechanism (SE-like), followed by processing the weighted features with spatial attention (similar to nonlocal net). This serial architecture offers two key advantages: (1) the frequency-domain priors provided by channel attention guide region selection in spatial attention, and (2) cross-dimensional gating (cross-dim gate) enables synergistic optimization of both attention mechanisms.

### 3.2 Intelligent thyroid diagnosis based on truth-value calibration

**Figure 5:**
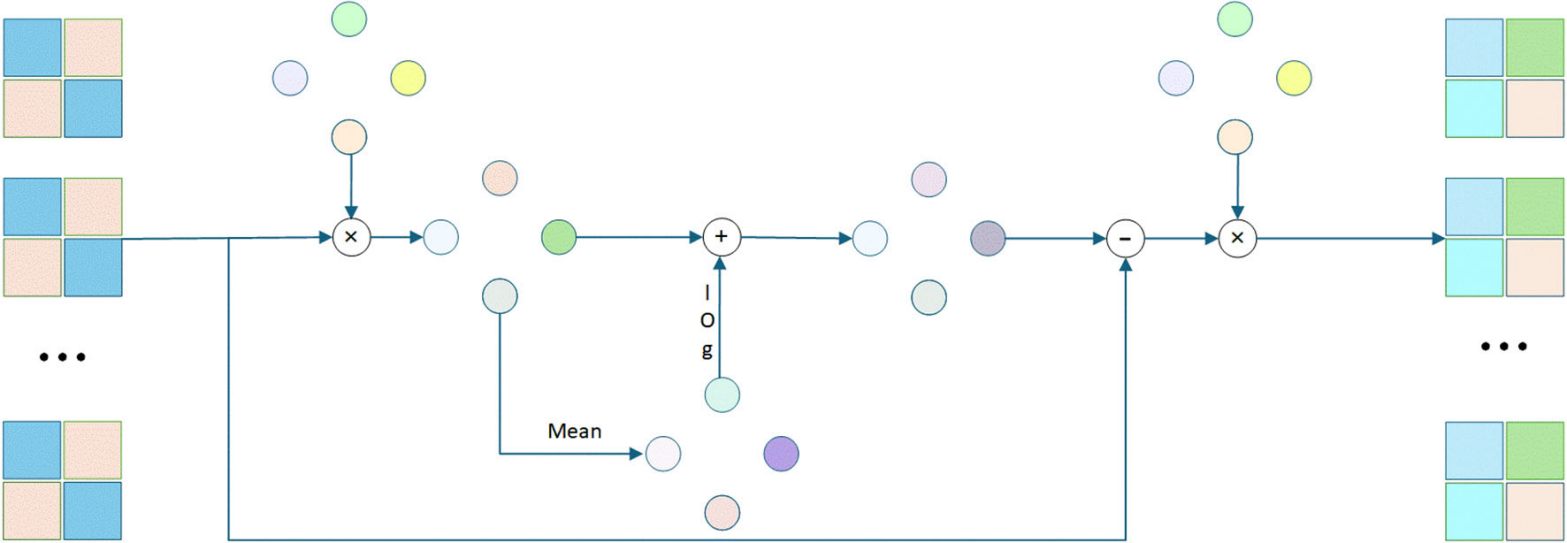
Truth-value calibration module.

To address the issue of inaccurate probability distribution predictions in models, this paper proposes a probability calibration method based on the truth-value calibration (TVC) algorithm. As shown in **Figure 5**, this method achieves precise calibration of the predicted probability distribution by iteratively optimizing the logit value output by the model. Through this iterative optimization process, our algorithm can effectively calibrate the probability distribution output by the model, making it more closely resemble the true distribution, thereby enhancing the model’s prediction accuracy and generalization ability, especially for scenarios with imbalanced classes or complex data distributions. The specific implementation process is as follows:

#### 3.2.1 Parameter initialization

Let the logit of the original output of the model be $Z\;=\;[Z_{1},Z_{2},\ldots ,Z_{c}]{\mathbb{R}}^{C}$, where C is the number of categories and where $Z_{i}$ represents the original logit value of the i category. Initialize the learnable weight matrix $W=\;[W_{1},W_{2},\ldots ,W_{c}]{\mathbb{R}}^{C}$, where $W_{i}$ is the calibration weight of the i category.

#### 3.2.2 Probability calculation and adjustment

In each iteration, the calibrated logit is calculated, as shown in Equation (1):

(1) $$Z'\;=\;Z⊙W=[Z_{1}W_{1},Z_{2}W_{2},\ldots ,Z_{c}W_{c}]$$

where ⊙ represents element-by-element multiplication. The calibrated probability distribution is subsequently calculated via the Softmax function, as shown in Equation (2):

(2) pi = ezi'∑j=1Cezj' , i = 1, 2, …, C

where $p_{i}$ is the calibration probability of the i category.

#### 3.2.3 Weight optimization

The objective function is defined as the KL divergence between the calibration probability and the real label distribution, as shown in Equation (3):

(3) $$L\;=\;D_{KL}(y||p)\;=\;\sum_{i=1}^{C}y_{i}\log(\frac{y_{i}}{p_{i}})$$

$y\;=\;[y_{1},y_{2},\ldots ,y_{c}]$ is the one-hot encoding of the real label. The weight W is iteratively updated via the gradient descent method, as shown in Equation (4):

(4) $$W\leftarrow W-\eta \nabla_{w}L$$

η is the learning rate.

#### 3.2.4 Iteration termination and output

When the loss function L converges or reaches the maximum number of iterations, the optimization process is terminated, and the final calibrated probability distribution P is output.

### 3.3 Intelligent thyroid diagnosis based on THMSNet

**Figure 6:**
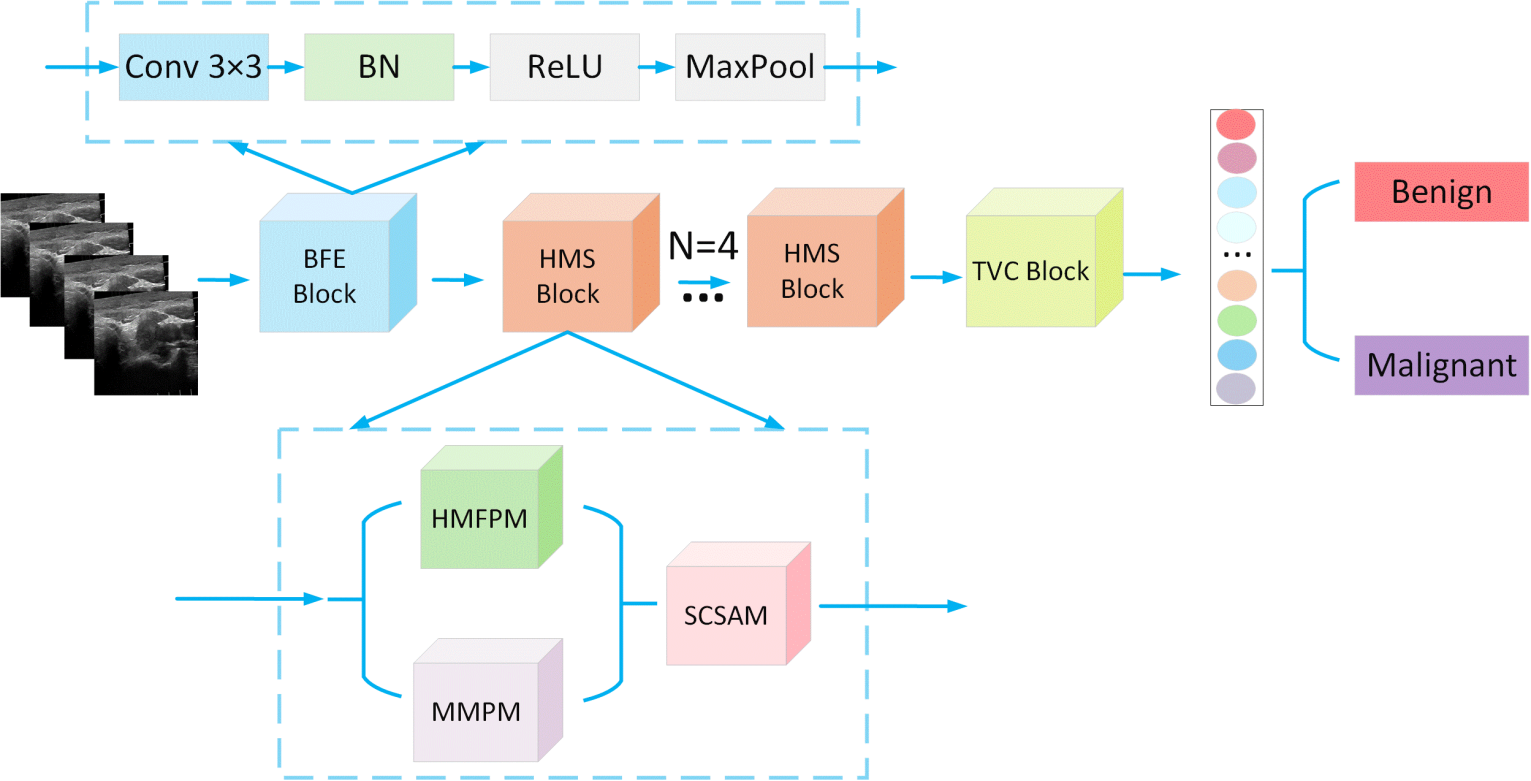
THMSNet.

As illustrated in **Figure 6**, THMSNet integrates five core components to achieve comprehensive and accurate thyroid nodule diagnosis: the basic feature extraction module (BFEM), the hierarchical multiscale feature pyramid (HMFP), the mamba-based multistage projection module (MMPM), the serial channel-spatial attention module (SCSAM), and the truth-value calibration (TVC) algorithm. The workflow of THMSNet is as follows:

The input thyroid ultrasound image first passes through the BFEM, which employs a four-stage “convolution-normalization-activation-pooling” cascade structure. This module captures fundamental local texture features of thyroid nodules, such as margin sharpness and internal echogenicity, using a 3×3 convolutional layer followed by batch normalization (BN), ReLU activation, and max pooling (MaxPool). This step ensures efficient computation while preserving essential morphological characteristics for subsequent processing.

The extracted features are then fed into the hierarchical multiscale feature pyramid (HMFP) module, which constructs a four-level pyramid with resolutions of H×W (global semantics), 2H×2 W (structural characteristics), 4H×4 W (boundary details), and 8H×8 W (microcalcifications). Adaptive alignment between levels is achieved via deformable convolution, whereas skip connections ensure seamless integration of multiscale features, enabling the model to handle nodules of varying sizes and complexities.

The multiscale features are processed by the Mamba-based multistage projection module (MMPM), which adopts a three-stage “projection-SSM-state update” pipeline. First, features are projected into the latent space via a 1×1 convolution. The Mamba block then models long-range dependencies via a 256-token sequence length and a Δ-parameterization mechanism to dynamically adjust the attention weights. Finally, a gating mechanism updates the hidden states, enhancing the model’s ability to capture the global context critical for malignancy assessment.

The serial channel–spatial attention module (SCSAM) further refines the features through a strict “channel–first” pipeline. Channel attention (SE-like) computes frequency-domain weights to highlight diagnostically relevant channels, followed by spatial attention (nonlocal net-inspired) to focus on key regions (e.g., irregular margins). Cross-dimensional gating synergistically optimizes both attention mechanisms, improving discriminative power for subtle malignant features.

The truth-value calibration (TVC) algorithm performs the final probability calibration by iteratively optimizing the logit values. Through KL divergence minimization and gradient descent-based weight updates, TVC aligns the model’s predicted probability distribution with pathological diagnostic standards. This calibration process significantly improves the model’s clinical applicability by reducing prediction bias and enhancing result interpretability.

### 3.4 Implementation details

The model was implemented using PyTorch and trained on an NVIDIA V100 GPU. We used a batch size of 32 and the AdamW optimizer with an initial learning rate of 1e-4. To enhance model robustness and prevent overfitting, we employed extensive data augmentation strategies including random horizontal and vertical flipping, rotation (± 15°), the addition of Gaussian noise, and contrast adjustment.

## 4 Results

### 4.1 Datasets

This study utilizes a high-quality public dataset from Kaggle for experimental analysis, focusing on benign and malignant thyroid classification. The dataset consists of a total of 7,288 thyroid images, including 3,282 benign cases (45.03%) and 4,006 malignant cases (54.97%). All the images were rigorously annotated and confirmed through pathological diagnosis. To ensure data consistency, the images underwent standardized preprocessing, including normalization and resizing, with a final uniform resolution of 224×224 pixels.

To evaluate the model’s performance, the dataset was randomly split into training and validation sets at an 8:2 ratio. The training set comprises 5,830 images (80% of the total data), whereas the independent validation set contains 1,458 images (20% of the total data). This stratified partitioning ensures a balanced distribution of benign and malignant cases in both sets, enabling a robust assessment of the model’s generalizability. The dataset is publicly available at https://www.kaggle.com/datasets/tingzen/thyroid-for-pretraining.

### 4.2 Evaluation indicators

This paper utilizes a range of evaluation metrics to comprehensively assess the performance of the proposed algorithm in classifying benign and malignant thyroid nodules. The specific metrics include accuracy (Acc), precision (Pre), recall (Recall), the F1 score, and the area under the receiver operating characteristic curve (AUROC). True positive (TP) refers to the number of multimolecular biomarker mutation samples correctly predicted by the model; true negative (TN) represents the number of multimolecular biomarker nonmutation samples correctly predicted by the model; false positive (FP) indicates the number of nonmutation samples that the model incorrectly predicts as mutations; and false negative (FN) represents the number of mutation samples that the model incorrectly predicts as nonmutation. The AUROC is the area under the receiver operating characteristic curve (ROC), with a value range of (0, 1). The closer the value is to 1, the better the model’s classification performance.

Acc refers to the ratio of the number of correctly classified samples in the test set to the total number of samples, and its formula is shown in Equation (5).

(5) $$\text{Acc }=\;\frac{\text{TP+TN}}{\text{TP+TN}+\text{FP+FN}}$$

Pre refers to the ratio of the number of true mutation samples in the test set to the number of predicted mutation samples, and its formula is shown in Equation (6):

(6) $$\text{Pre }=\;\frac{\text{TP}}{\text{TP}+\text{FP}}$$

Recall refers to the ratio of the number of samples correctly predicted as mutations to the number of true mutations in the test set, and its formula is shown in Equation (7).

(7) $$\text{Recall}=\;\frac{\text{TP}}{\text{TP}+\text{FN}}$$

The F1 score is the harmonic mean of Pre and Recall, and its formula is shown in Equation (8).

(8) F1-score= 2×Pre×RecallPre+Recall

### 4.3 Comparison of different pyramid architecture depths in the intelligent diagnosis of thyroid cancer

**Figure 7:**
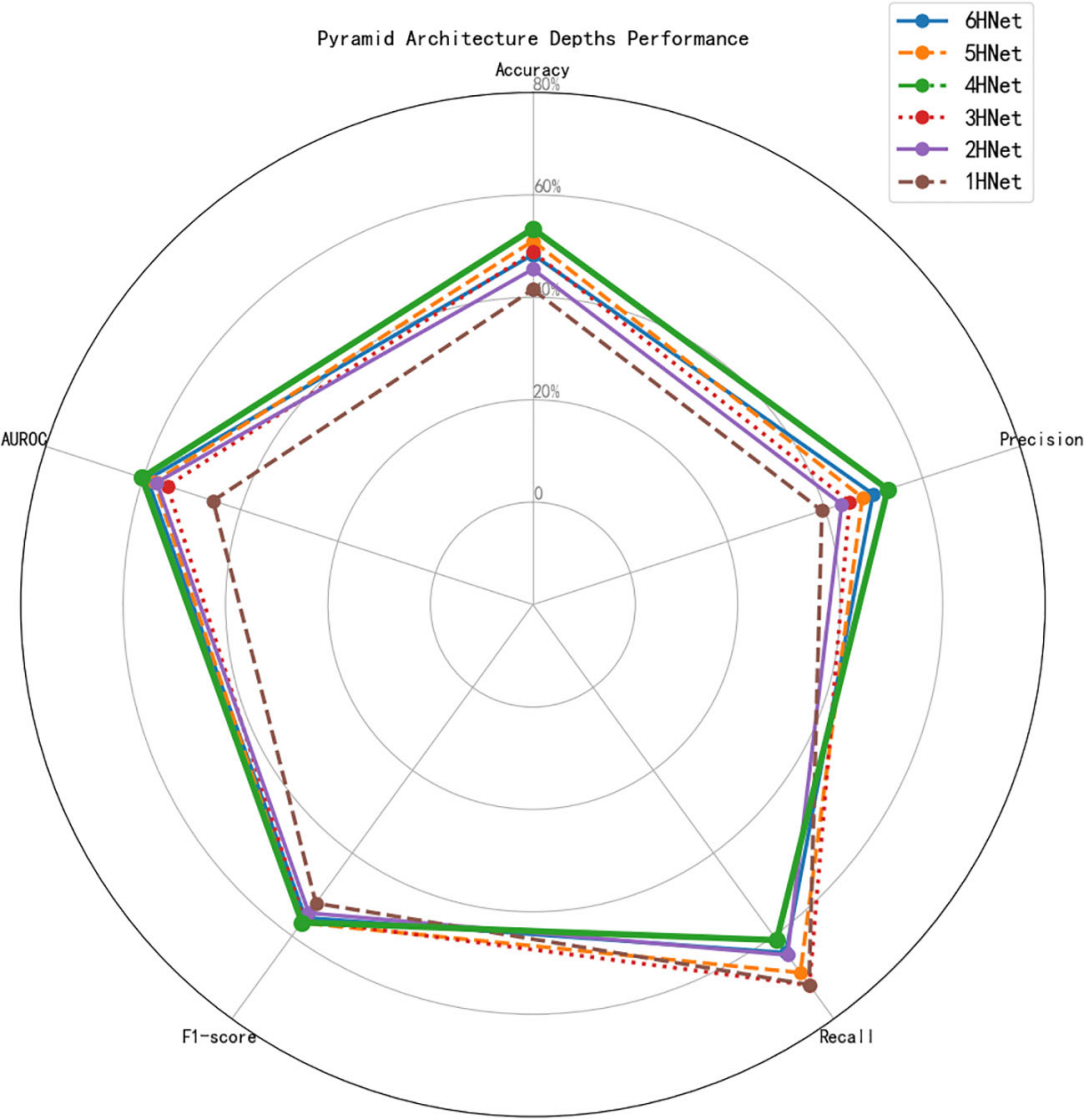
Comparison of pyramid architecture depths across different metrics.

The experimental results demonstrate that the depth of the pyramid architecture significantly impacts diagnostic performance in thyroid cancer classification. As shown in **Table 1** and **Figure 7**, 4HNet achieves the best overall performance, with the highest accuracy (73.39%), precision (72.86%), and AUROC (80.24%), while maintaining a balanced recall (80.99%) and F1 score (76.71%). Deeper networks (5HNet and 6HNet) exhibit improved recall (88.85% and 84.04%, respectively) but suffer from lower precision, suggesting overfitting to malignant cases. Conversely, shallower architectures (1HNet–3HNet) result in high recall (up to 91.89% for 3HNet) but significantly lower precision and AUROC, indicating poor generalizability. Compared with 4HNet, the 2HNet model strikes a middle ground but underperforms. These findings suggest that moderate depth (4 layers) optimizes the trade-off between feature extraction and computational efficiency, making it the most suitable choice for thyroid nodule classification.

**Table 1:** Comparison of different pyramid architecture depths.

Model	Accuracy (%)	Precision (%)	Recall (%)	F1-score (%)	AUROC (%)
6HNet	68.34	69.84	84.04	75.59	79.12
5HNet	70.99	67.66	88.85	**76.82**	77.70
4HNet	**73.39**	**72.86**	80.99	76.71	**80.24**
3HNet	68.86	65.02	**91.89**	76.16	75.03
2HNet	65.50	63.42	84.57	74.44	77.22
1HNet	61.52	59.34	91.76	72.08	65.65

Bold values indicate the best performance.

### 4.4 Comparison of different attention mechanisms in the intelligent diagnosis of thyroid cancer

**Figure 8:**
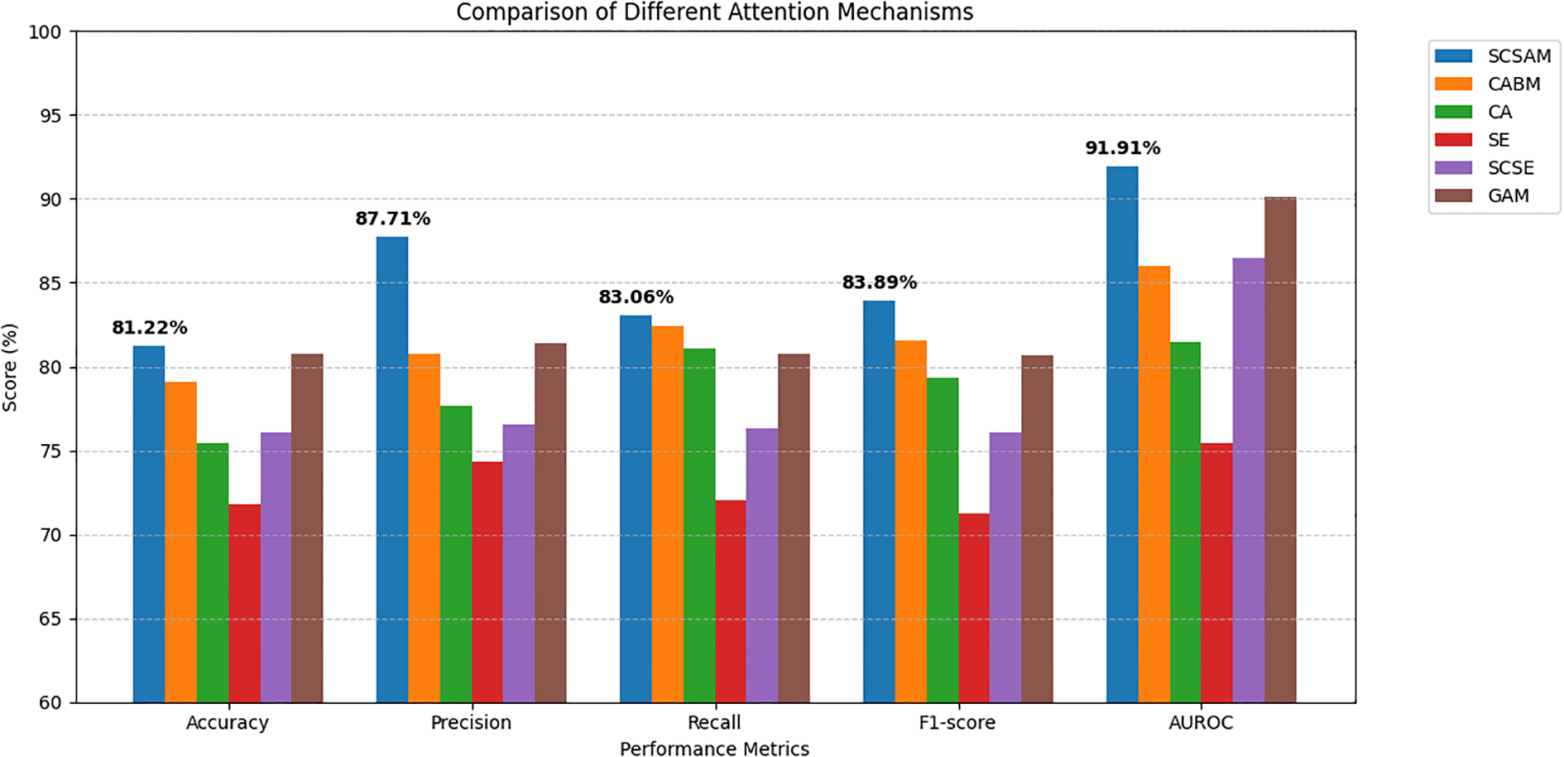
Comparison of different attention mechanisms.

The experimental results demonstrate the superior performance of the proposed serial channel-spatial attention module (SCSAM) over other attention mechanisms in thyroid cancer diagnosis. As shown in **Table 2** and **Figure 8**, the SCSAM achieves the highest scores across all the evaluation metrics, including accuracy (81.22%), precision (87.71%), recall (83.06%), F1 score (83.89%), and AUROC (91.91%). This outstanding performance can be attributed to its effective serial integration of channel and spatial attention mechanisms, which enables more comprehensive feature extraction and refinement. The comparative analysis reveals that while GAM (80.75% accuracy) and CBAM (79.11% accuracy) yield competitive results, they fall short of SCSAM’s performance, particularly in terms of precision and AUROC metrics. Other mechanisms, such as SCSE, CA, and SE, exhibit progressively weaker performance, with SE showing the lowest scores across all the metrics (71.83% accuracy). These results clearly indicate that the sequential processing of channel and spatial information in the SCSAM provides significant advantages for thyroid nodule classification, making it the most suitable attention mechanism for this medical imaging task. The remarkable AUROC score of 91.91% highlights SCSAM’s strong discriminative ability in distinguishing malignant from benign thyroid nodules.

**Table 2:** Comparison of different attention mechanisms.

Model	Accuracy (%)	Precision (%)	Recall (%)	F1-score (%)	AUROC (%)
SCSAM	**81.22 ± 0.03**	**87.71 ± 0.05**	**83.06 ± 0.03**	**83.89 ± 0.05**	**91.91 ± 0.06**
CABM (17)	79.11	80.78	82.39	81.58	86.02
CA (19)	75.44	77.63	81.04	79.30	81.49
SE (16)	71.83	74.31	72.05	71.22	75.47
SCSE (18)	76.06	76.52	76.31	76.04	86.50
GAM (20)	80.75	81.36	80.78	80.67	90.15

Bold values indicate the best performance.

### 4.5 Comparison of different existing models and methods for intelligent thyroid cancer diagnosis

**Figure 9:**
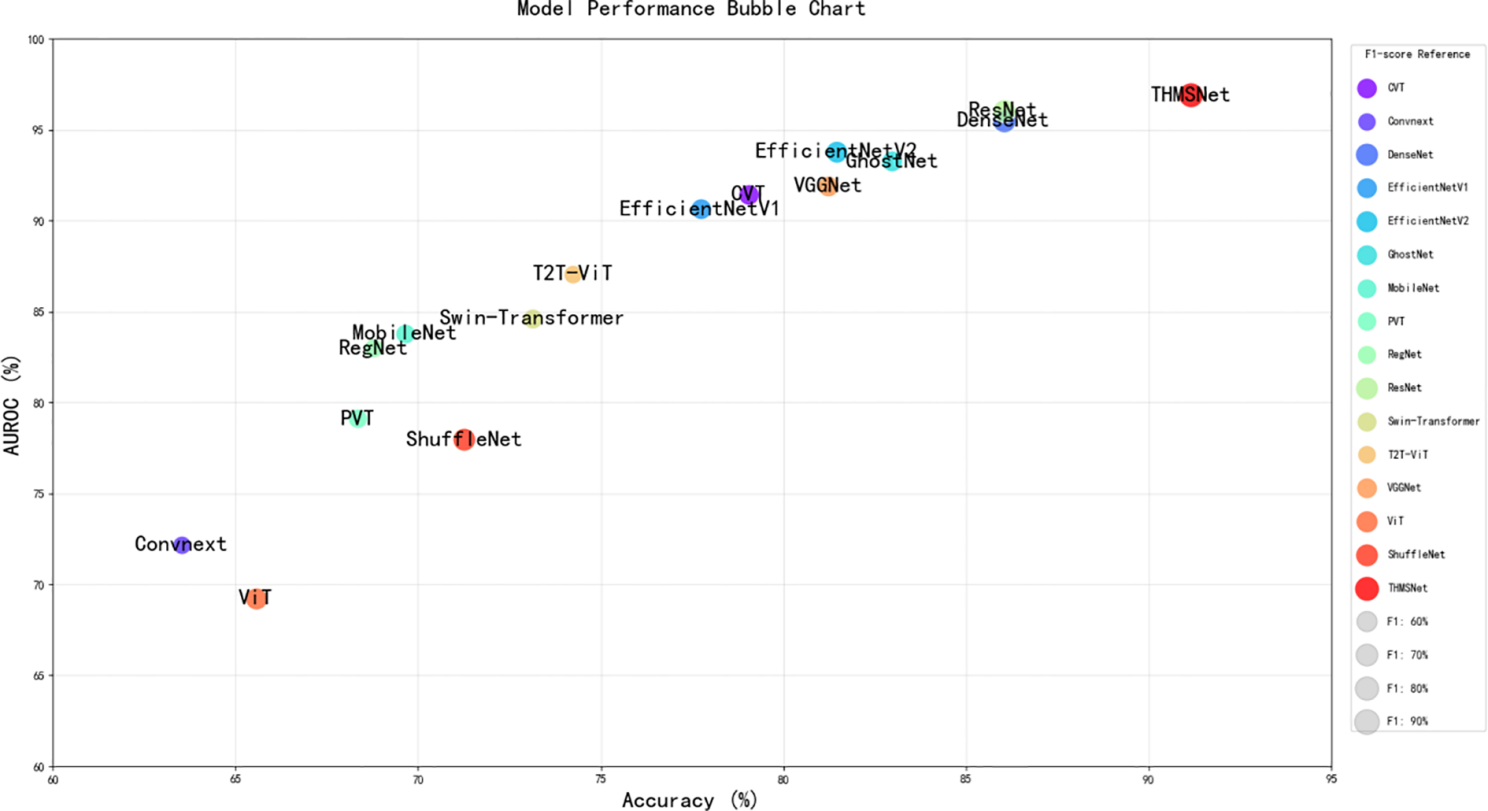
Model performance bubble chart.

As demonstrated in **Table 3**, **Figure 9** and **Table 4**, our comprehensive evaluation reveals significant performance variations among existing deep learning models and published methods for thyroid cancer diagnosis. **Table 3** shows that THMPNet establishes a new benchmark with exceptional metrics (91.15% accuracy, 91.94% F1 score, and 96.92% AUROC), outperforming all other architectures, including ResNet (86.03% accuracy) and DenseNet (95.50% AUROC). Traditional models such as VGGNet (81.22% accuracy) and transformer-based approaches (CVT: 79.04%) exhibit competitive but inferior performance. Notably, **Table 4** highlights that THMPNet surpasses all prior literature results by substantial margins, achieving >8% higher accuracy than Moran’s method (86.22%) and >17% improvement over Wang’s best-reported accuracy (74.69%). The comparative analysis highlights two critical findings: (1) architectural innovation (THMPNet) delivers superior diagnostic capability compared with conventional CNNs/transformers, and (2) existing published methods generally underperform against modern deep learning models, with the highest literature AUROC (Moussa et al: 74.00%) being 22.92% lower than that of THMPNet (96.92%). These results validate the clinical potential of THMPNet while revealing limitations in current state-of-the-art approaches for detecting thyroid malignancy.

**Table 3:** Comparison of different existing models.

Model	Accuracy (%)	Precision (%)	Recall (%)	F1-score (%)	AUROC (%)
CVT (28)	79.04	64.99	61.98	62.98	91.46
Convnext (29)	63.53	55.99	49.87	50.37	72.17
DenseNet (30)	86.03	76.78	83.56	79.27	95.50
EfficientNetV1 (31)	77.73	67.20	62.36	63.82	90.65
EfficientNetV2 (32)	81.44	70.28	71.75	70.84	93.81
GhostNet (33)	82.97	75.42	62.61	64.39	93.27
MobileNet (34)	69.65	62.09	55.49	57.33	83.79
PVT (35)	68.34	69.84	54.04	55.90	79.12
RegNet (36)	68.78	62.53	52.45	54.01	83.00
ResNet (37)	86.03	75.46	79.48	77.10	96.07
Swin-Transformer (38)	73.14	66.22	57.55	58.70	84.63
T2T-ViT (39)	74.24	49.29	52.80	50.95	87.07
VGGNet (40)	81.22	67.71	63.06	63.89	91.91
ViT (41)	65.57	64.17	82.38	72.14	69.23
ShuffleNet (42)	71.26	67.58	90.37	77.33	77.97
THMSNet	**91.15 ± 0.05**	**90.64 ± 0.03**	**93.28 ± 0.02**	**91.94 ± 0.04**	**96.92 ± 0.03**

Bold values indicate the best performance.

**Table 4:** Comparison of different existing methods.

Model	Accuracy (%)	Precision (%)	Recall (%)	F1-score (%)	AUROC (%)
Wang et al (43)	66.81	–	–	–	63.71
Wang et al (43)	74.69	–	–	–	71.27
Bai et al (44)	76.40	–	–	–	–
Thomas et al (45)	81.50	–	–	–	–
Ma et al (46)	83.02	–	–	–	–
Ma et al (47)	83.02	–	–	–	–
Mei et al (48)	53.00	–	–	–	–
Moran et al (49)	86.22	–	–	–	–
Moussa et al (50)	81.83	–	–	–	74.00
THMSNet	**91.15 ± 0.05**	**90.64 ± 0.03**	**93.28 ± 0.02**	**91.94 ± 0.04**	**96.92 ± 0.03**

Bold values indicate the best performance.

### 4.6 Performance on small nodules

To evaluate the model’s performance more comprehensively, we performed a stratified analysis based on nodule size. This analysis was conducted to assess how THMSNet performs on thyroid nodules of different sizes, particularly addressing the challenge of detecting small nodules (<1 cm), which is crucial for early-stage malignancy detection.

The results presented in **Table 5** indicate that the model’s performance on small nodules (<1 cm) is suboptimal compared to larger nodules. Small nodules often represent early-stage malignancies, and the model struggles to capture their fine-grained features due to resolution constraints and pooling operations. However, the model performs significantly better on larger nodules, with both accuracy and AUROC improving as the nodule size increases.

**Table 5:** Stratified results by nodule size.

Nodule size	Accuracy (%)	Precision (%)	Recall (%)	F1-Score (%)	AUROC (%)
<1 cm	75.30	72.45	70.18	71.20	85.45
1–2 cm	90.11	89.24	91.56	90.40	94.85
>2 cm	92.78	91.75	93.65	92.69	96.15

### 4.7 Ablation experiment

**Figure 10:**
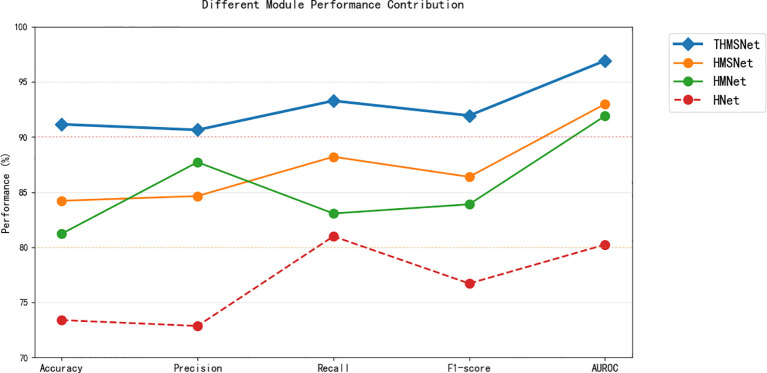
Performance contributions of different modules.

**Figure 11:**
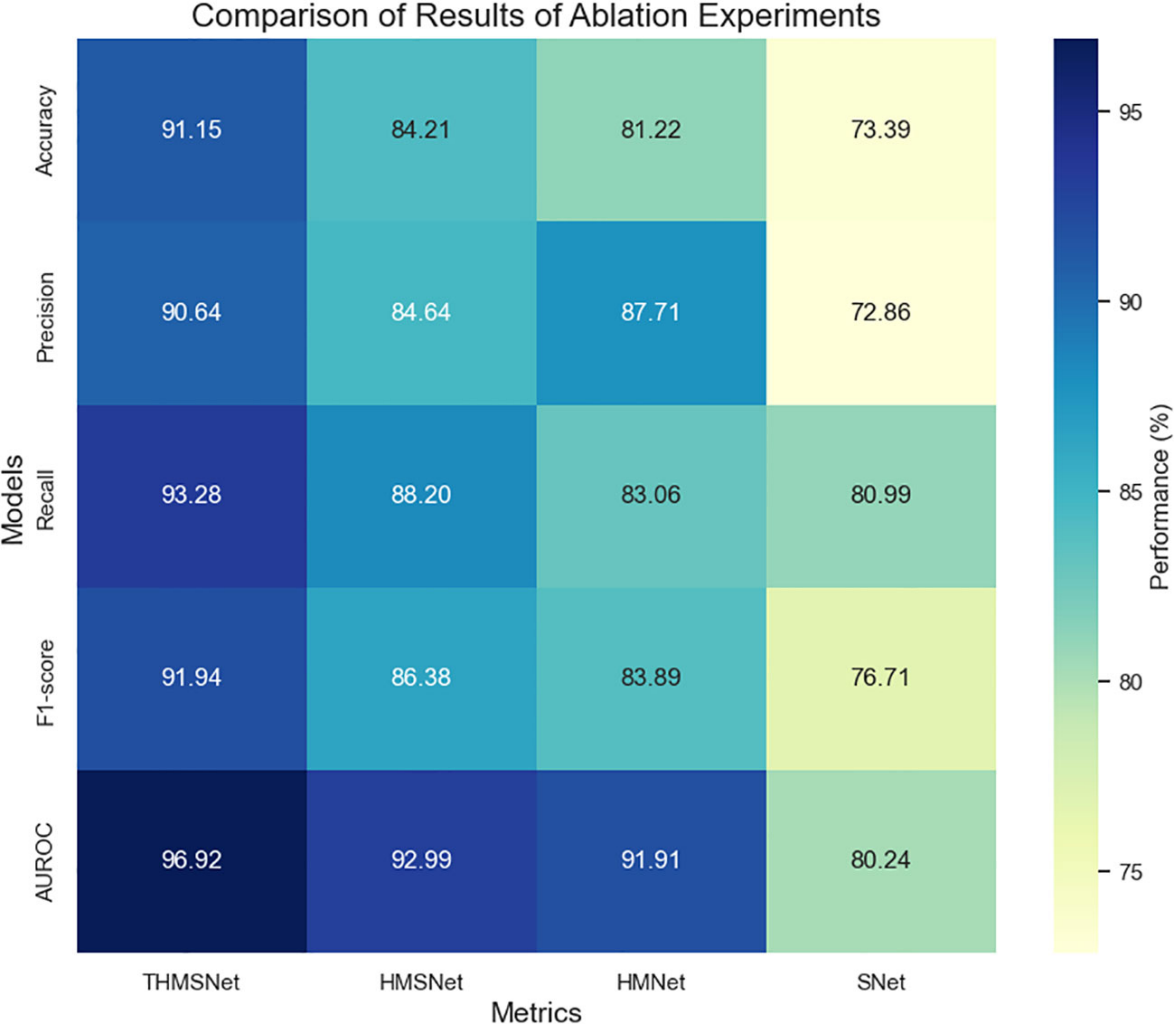
Comparison of the results of the ablation experiments.

The ablation studies presented in **Table 5**, **6**, **Figure 10** and **Figure 11** demonstrate the progressive performance improvements achieved by sequentially integrating key components into THMSNet. The pyramid architecture (HNet) establishes a strong baseline (73.39% accuracy) by enabling multiscale feature extraction, which is critical for analyzing thyroid nodules of varying sizes. The addition of the Mamba module (HMNet) yields the most substantial gain (+7.83% accuracy), validating its effectiveness in modeling long-range spatial dependencies through selective state space mechanisms. The incorporation of the SCSAM attention module (HMSNet) provides further refinement (+2.99% accuracy), with its serial channel-spatial attention mechanism proving particularly adept at enhancing discriminative local features. Finally, the truth calibration component (THMSNet) delivers the most clinically significant improvement (+6.94% accuracy) by aligning model predictions with diagnostic standards while also achieving the highest AUROC (96.92%), demonstrating exceptional malignancy discrimination capability. This systematic evaluation confirms that each module addresses distinct challenges in medical image analysis: the pyramid structure handles scale variation, Mamba captures the global context, SCSAM optimizes local features, and truth value calibration ensures clinical relevance. The full integration of these complementary components in THMSNet achieves state-of-the-art performance (91.15% accuracy), establishing a new benchmark for thyroid nodule classification that simultaneously advances technical innovation and clinical applicability.

**Table 6:** Comparison of the results of the ablation experiments.

Model	Accuracy (%)	Precision (%)	Recall (%)	F1-score (%)	AUROC (%)
THMSNet	**91.15 ± 0.05**	**90.64 ± 0.03**	**93.28 ± 0.02**	**91.94 ± 0.04**	**96.92 ± 0.03**
HMSNet	84.21	84.64	88.20	86.38	92.99
HMNet	81.22	87.71	83.06	83.89	91.91
HNet	73.39	72.86	80.99	76.71	80.24

Bold values indicate the best performance.

### 4.8 Generalization on an external cohort or patient-level cross-validation

To evaluate the generalization capability of THMSNet in real-world clinical settings, we conducted external validation on a completely separate dataset, distinct from the original training data. The external dataset used for validation consists of thyroid ultrasound images from a different source containing a total of 1,200 thyroid nodules, annotated with pathological diagnoses (600 benign, 600 malignant).

We performed patient-level cross-validation on this external dataset, ensuring that all images from a given patient were either in the training or testing set, but not in both. This approach mirrors a real-world scenario where the model needs to generalize to new patients with different characteristics.

As shown in **Table 7**, THMSNet achieved a mean accuracy of 90.05% and a mean AUROC of 93.46% on the external validation dataset. These results demonstrate the model’s strong ability to generalize to new, unseen data from a different clinical source. Performance of THMSNet on the external validation dataset. The mean accuracy and AUROC are reported with their respective standard deviations, confirming the model’s robustness and generalization to new patient data.

**Table 7:** The performance of THMSNet on the external cohort.

Metric	Mean value (%)	Confidence interval (%)	Standard deviation (%)
Accuracy	90.05	87.95 - 92.15	± 2.1%
AUROC	93.46	92.16 - 94.76	± 1.3%

These findings indicate that THMSNet is capable of performing well not only on the original training dataset but also when applied to external cohorts, further enhancing its clinical applicability. This validation supports the use of THMSNet as a reliable tool for thyroid nodule diagnosis in diverse clinical environments.

### 4.9 Analysis of predicted probabilities and brier score

**Figure 12:**
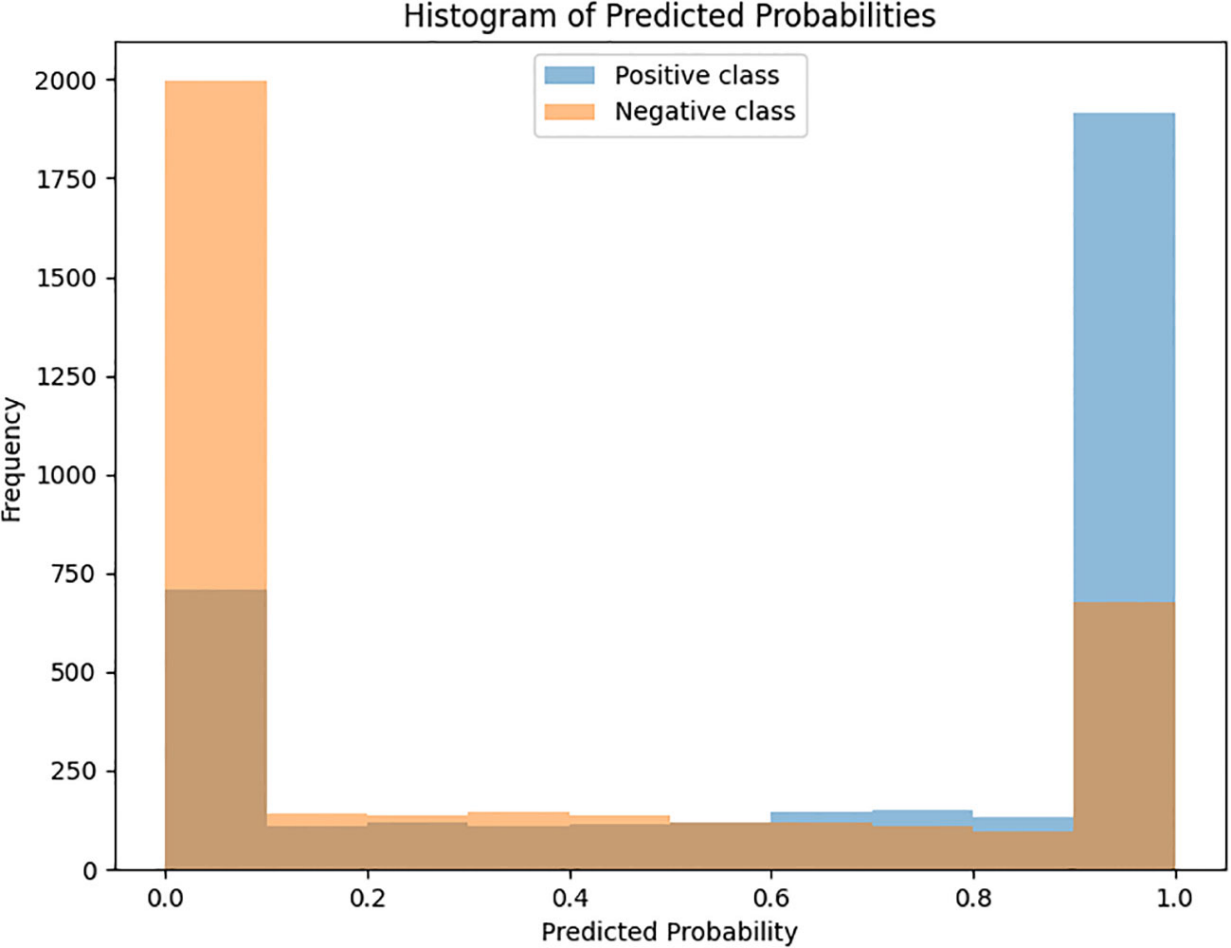
The distribution of predicted probabilities for both the positive and negative classes.

As part of our evaluation of the model’s performance, we analyzed the predicted probabilities for thyroid nodule classification. The Histogram of Predicted Probabilities clearly shows a stark separation in the predicted probabilities for the positive and negative classes. The positive class is highly concentrated around a probability of 1, while the negative class is predominantly concentrated near 0. As shown in **Figure 12**, the predicted probability distributions of the positive and negative classes exhibit a clear separation, with most malignant cases assigned high probabilities and benign cases concentrated near zero.

The Brier Score for the model’s predictions is 0.2632. As shown in **Figure 2**, histogram of predicted probabilities shows the distribution of predicted probabilities for both the positive and negative classes, highlighting the model’s tendency to assign extreme probabilities. For a more clinically relevant model, a Brier score closer to 0.25 would indicate better-calibrated probabilities. This score would suggest that the model is confident, but not excessively so, and that its predictions align more closely with the actual labels. Future work could focus on methods for calibrating the model’s predictions to achieve a lower Brier score, such as using techniques like Platt scaling or isotonic regression.

## 5 Discussion

The proposed THMSNet demonstrates significant advancements in thyroid nodule diagnosis by addressing three critical challenges in medical image analysis: insufficient multiscale feature extraction, weak long-range dependency modeling, and misalignment between model predictions and clinical standards. The hybrid architecture, which combines a pyramid structure for local feature extraction with Mamba for global context modeling, achieves robust and multiscale feature representations capable of capturing both local texture patterns and global contextual dependencies, as evidenced by the 91.15% accuracy and 96.92% AUROC. The SCSAM attention mechanism further enhances discriminative power by hierarchically integrating channel and spatial attention, outperforming existing methods such as CBAM and GAM (**Table 2**). Additionally, the truth-value calibration algorithm bridges the gap between model outputs and pathological standards, improving clinical applicability. These innovations collectively enable THMSNet to outperform state-of-the-art models, including ResNet and DenseNet, while maintaining computational efficiency, making it a promising tool for assisting doctors in diagnosing thyroid conditions.

Despite its strengths, THMSNet has several limitations that warrant further investigation and improvement.

### 5.1 Performance on small nodules

The model’s performance on small nodules (<1 cm) remains suboptimal, as observed in the stratified results presented in the 4.6 section. This limitation is particularly significant because small nodules often indicate early-stage malignancies, and their timely detection is crucial for improving patient outcomes. The lower accuracy and recall for small nodules can be attributed to the challenge of capturing fine-grained features, which is often hindered by resolution constraints and pooling operations inherent in the model architecture.

While the model performs well on larger nodules, further enhancements are needed to improve detection for smaller ones. Potential solutions include the use of higher-resolution input images or multi-scale patch-based approaches, which could enhance the model’s ability to capture smaller, detailed features. Exploring such methods in future work may lead to improvements in detecting early-stage malignancies, ultimately making the model more robust for clinical applications (51).

### 5.2 Dataset limitations and generalizability

The present study is based solely on a single public dataset from Kaggle, which contains 7,288 thyroid ultrasound images. While this dataset provides a valuable and standardized benchmark, relying exclusively on one source raises concerns regarding the robustness and generalizability of THMSNet across diverse populations, ultrasound devices, and clinical environments. As highlighted by Baima et al. (14), multicenter validation is essential to ensure consistent performance across varied acquisition protocols and demographic distributions. Future research should therefore incorporate multicenter or in-house datasets with broader patient characteristics, including differences in age, sex, and ethnicity, to rigorously evaluate real-world applicability. In addition, advanced strategies such as domain adaptation (52) and federated learning (53) may be explored to mitigate potential domain shifts while preserving patient privacy, further enhancing the generalizability of the model.

### 5.3 Label consistency and noisy clinical data

While the truth-value calibration (TVC) algorithm significantly improves the model’s calibration, real-world clinical environments often present challenges such as noisy or ambiguous cases, which may affect diagnostic accuracy. To address this, incorporating uncertainty quantification techniques could further enhance the robustness of the model. Methods like Bayesian deep learning and Monte Carlo dropout are particularly valuable in these contexts.

Bayesian deep learning (54) provides a natural framework for quantifying uncertainty by placing distributions over the model’s weights. This allows the model to output not only predictions but also a measure of confidence in those predictions. Such uncertainty estimates are crucial for handling ambiguous cases, where the model may be less certain about its classification, potentially indicating areas where clinician intervention or further testing is needed.

Additionally, Monte Carlo (55) dropout is also a popular technique that introduces stochasticity during both training and inference by randomly dropping units from the network. This method can be employed to approximate the uncertainty in the model’s predictions. By performing multiple stochastic forward passes during inference, we can obtain a distribution of predictions, which can then be used to estimate the uncertainty associated with each diagnosis.

Incorporating these techniques into THMSNet could improve its ability to handle uncertain or noisy data, providing more reliable predictions in clinical practice. Future work will explore the integration of such methods to ensure that the model not only delivers accurate results but also quantifies its confidence, which is essential for making informed clinical decisions.

### 5.4 Integration with clinical workflows

Although THMSNet demonstrates robust diagnostic performance, its practical integration into clinical workflows requires careful consideration. For seamless adoption in radiology, it is essential that THMSNet is compatible with existing Picture Archiving and Communication Systems (PACS), enabling smooth data exchange between the diagnostic model and radiological platforms. By integrating with PACS, THMSNet can automate thyroid nodule classification alongside ultrasound images, thus streamlining the workflow and enhancing productivity.

Another important factor for clinical deployment is the inference speed of the model. Ensuring that THMSNet can provide real-time diagnostic support is crucial for its practical use. This can be achieved by optimizing the model for faster inference times through methods such as model quantization or hardware acceleration, ensuring it operates efficiently in clinical settings without compromising diagnostic accuracy.

Furthermore, the interpretability of the model’s outputs is critical for gaining clinician trust and facilitating informed decision-making. THMSNet’s outputs should be accompanied by visual aids, such as heatmaps or confidence scores, to clearly indicate the regions of the image that contributed to the classification, thereby helping clinicians understand the model’s reasoning. These interpretability tools will allow radiologists to make more informed decisions, combining their clinical expertise with the insights provided by the AI system.

To fully realize its potential, THMSNet must seamlessly integrate into existing clinical workflows, offering real-time, interpretable results that align with radiologists’ daily practices. Future work should prioritize optimizing inference speed, ensuring PACS compatibility, and enhancing the interpretability of outputs, thereby making THMSNet a valuable and efficient tool in clinical settings.

### 5.5 Rare nodule subtypes

Rare thyroid subtypes, which often share overlapping features with benign nodules, present a significant challenge in medical imaging. These subtypes are not only underrepresented in the training data but may also be difficult for the model to classify accurately due to their subtle and atypical characteristics. Few-shot learning methods, such as meta-learning, can help the model adapt to these rare cases without requiring large amounts of labeled data.

Another potential solution is synthetic data augmentation, where Generative Adversarial Networks (GANs) or similar techniques are used to generate synthetic images that resemble rare thyroid subtypes. This augmented data could help train THMSNet to recognize the distinguishing features of these rare nodules, improving its diagnostic accuracy in clinical settings. By integrating few-shot learning and synthetic augmentation, THMSNet could be better equipped to handle the variability and complexity of rare thyroid subtypes, ultimately expanding its applicability in diverse clinical environments.

Addressing these limitations in future research will not only enhance the performance of THMSNet but also accelerate its translation into clinical practice, ultimately improving patient outcomes in thyroid nodule diagnosis.

## 6 Conclusion

This study presents THMSNet, a robust AI-assisted diagnostic system for thyroid nodules that synergistically integrates a pyramid architecture, the Mamba module for long-range dependency modeling, a novel serial channel-spatial attention mechanism (SCSAM), and a truth-value calibration (TVC) algorithm. The integrated model achieves state-of-the-art performance (91.15% accuracy and 96.92% AUROC) by effectively addressing key challenges in medical image analysis. As a clinical decision-support tool, it provides both quantitative analysis and visual interpretation to assist radiologists. Future work will focus on: developing lightweight versions of THMSNet via pruning and quantization for efficient clinical deployment; conducting large-scale multicenter validation to verify robustness and generalizability; exploring semi-supervised learning to mitigate label noise; and integrating the system into hospital PACS for real-time use in thyroid clinics to facilitate clinical translation.

## Data Availability

The original contributions presented in the study are included in the article/supplementary material. Further inquiries can be directed to the corresponding author.
